# Comparison between a fitness tracker (Equimetre^TM^) and standard base-apex electrocardiography in dromedary camels

**DOI:** 10.3389/fvets.2022.963732

**Published:** 2023-01-12

**Authors:** Taleb Al Khamis, Turke Shawaf, Adel Almubarak, Mohammed Ali Al-Ali

**Affiliations:** Department of Clinical Sciences, College of Veterinary Medicine, King Faisal University, Al-Ahsa, Saudi Arabia

**Keywords:** camel, electrocardiograms, Equimetre^TM^, standard, tracker

## Abstract

**Background:**

Personalized healthcare technology has grown explosively through the use of portable and smart monitoring devices for diagnosis. The objective of this study was to determine the practicality and usability of the Equimetre^TM^ fitness tracker on camels in comparison to the standard base-apex system in normal and clinical cases.

**Methods:**

Five apparently healthy adult camels, five clinical adult cases and two clinical calves were enrolled in this study. The camels were equipped with two monitoring systems: Equimetre^TM^ and a standard base-apex electrocardiogarphy. Each tracing was evaluated for the normal ECG variable's measure, including heart rate beats per min, P-R, QRS, R-R, Q-T, S-T intervals, and P-R and S-T segments in seconds. The amplitudes for P, Q, R, S, and T-peaks were evaluated in millivolts.

**Results:**

Equimetre^TM^ showed stability on ECG tracing with less movement artifacts compared with the standard base-apex system. Different polarities were observed for the P-waves and T-waves between the standard base-apex system and Equimetre^TM^. Both devices showed perfect agreement for heart rate (ICC = 1.00, *P* ≥ 0.0001, 95% = 1.00–1.00) in healthy and clinical adults. A good correlation was observed for the R-R interval between the devices in healthy and clinical adults. A moderate correlation was observed between the devices for Q-peak in clinical adults, with no correlation in clinical calves.

**Conclusions:**

This study demonstrated acceptable ECG measurements between the standard base-apex and Equimetre^TM^ device. This suggests that Equimetre^TM^ could be a useful device in camels for initial electrocardiographic examinations in remote areas such as deserts.

## 1. Introduction

Waller (1) was the first to find electrocardiograms from humans, horses, dogs, cats, and rabbits using the capillary electrometer. Since then, aside from the huge literatures on animal experimentation, modest literature has accumulated on useful electrocardiography (2). Currently, personalized healthcare technology has grown explosively using portable and smart monitoring devices for diagnosis (3–5). Telemedicine techniques and tools provide professionals with rapid access to health monitoring and care implementation (6–8). Wearable medical devices play an essential role in continuous real-time medical data generation, helping in track metabolic status, diagnosis, and treatment (9). Teleconsultation using mobile health and wearable devices for monitoring animal health in veterinary medicine has become more effective, especially in areas with limited care access or lack of resources (10).

The applications of wearable devices are wide, ranging from biomedical to healthcare monitoring systems (11). Moreover, wearable sensor-based monitors enable the understanding of animals' activities to assess their safety and welfare, in addition to emotional behavior (12). New wearable medical devices in the field of cardiology have been increasingly used to measure heart activity, rate, and rhythm (13). The trend in the development of electrocardiogram (ECG) wireless body multi-functional sensors has shown accurate and reliable solutions for heart rhythm on long-term monitoring (14). The presence of several commercially available wearable and portable ECGs devices in human and veterinary fields has been demonstrated in recent studies on humans (15, 16), dogs (17), ruminants (18), and horses (19, 20).

Interest in camel's (*Camelus Dromedarius*) has increased dramatically over the past decades as a result of the innovation of many sports activities related to this animal. Thus, there has been an increasing need to provide the best medical care for this species (21).

The method for assessing camels heart rhythm through ECG has traditionally used the base-apex system (22, 23). Based on our knowledge obtaining a good quality ECG using an inexpensive, user-friendly, and digitalized device for data acquisition and evaluation has not been validated in camels. Therefore, in this study, we used the equine fitness tracker Equimetre^TM^ (Arinoneo, Paris, France), which has been validated in horses (24). This fitness tracker designed for a daily exercise monitoring by owners, riders and trainers providing a comprehensive data acquisition of speed, distance, stride frequency, and stride length, in addition to its capability of recording heart rate and ECG. The objective of this study was to determine the practicality and usability of the fitness tracker Equimetre^TM^ on camels and to compare it with the standard base-apex system in healthy and clinical cases.

## 2. Materials and methods

### 2.1. Animals

A total of five apparently healthy adult camels (*Camelus dromedaries*) owned by King Faisal University, Camel Research Center, five clinical adult cases [two male patients complaining of paraphimosis, one male patient with inflamed dulla (soft palate), one male patient with an open mandibular wound, and one female patient with an ear infection], and two clinical calves with cloudy eyes admitted to King Faisal Veterinary Teaching Hospital were enrolled in this study. The body score condition (BSC) of the camels was determined based on a previous report and rated from 1 (very thin) to 5 (fat) (25). Apparently healthy camels were determined based on a full clinical examination and history. Furthermore, all healthy camels were maintained in single-stall barns with free access to feed and water according to the standard care of King Faisal University Research and Training Station, Al Hasa.

### 2.2. Equipment

**Figure 1:**
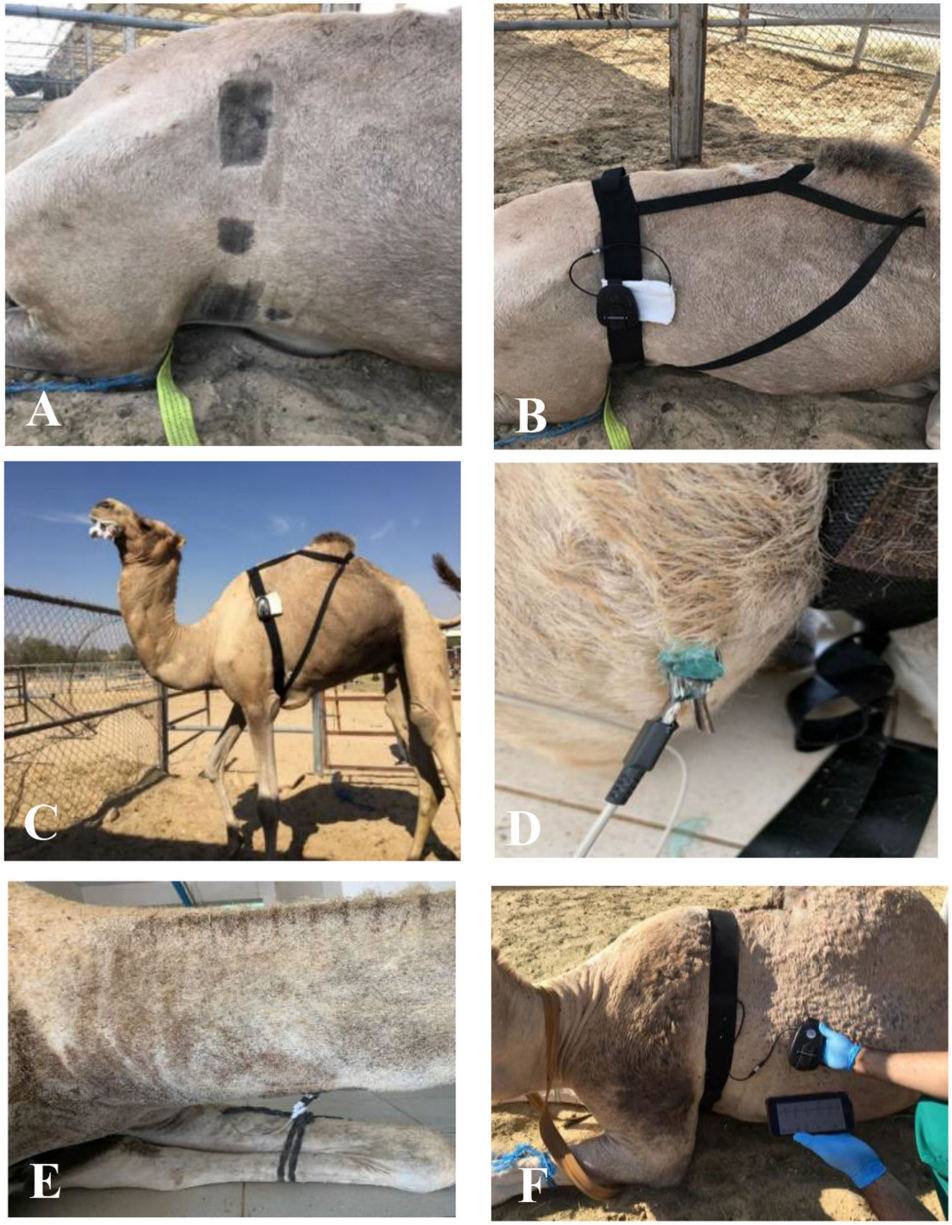
Positioning electrodes of both devices on camels **(A)** Shaving area of electrodes **(B)** Fixing Equimetre Vet **(C)** Girth after modification **(D)** Negative standard base-apex electrode **(E)** Positive standard base-apex electrode **(F)** Display of live ECG from Equimetre^TM^.

The camels were equipped with two monitoring systems: an Equimetre^TM^ (Equimetre^TM^, Arinoneo Paris, France) consisting of a two-electrode sensor on an elastic girth and a standard base-apex ECG (BM7 VET; Bionet, Republic of Korea) with surface electrodes attached to the skin using alligator clips. All camels were restrained in the sternal position unsedated. The electrodes were placed after shaving the electrode area (Figure 1A) using a Panasonic hair trimmer machine (Panasonic, hair trimmer ER2051, China) in three apparently healthy and two clinical adult camels. However, no shavings were made for two apparently healthy camels, three clinical adult camels, and two clinical calves. The first electrode of the Equimetre^TM^ girth was positioned on the left side of the chest, just behind the elbow joint, caudal to the olecranon at the cardiac apex. The second electrode was positioned two-thirds of the way down the thoracic girth (about 20 cm ventral to thoracic vertebrae). Modification on the girth was only made to fit the camel morphemically to secure the girth for better ECG reading (Figure 1B, C).

The left arm electrode (positive) of the standard base-apex was placed above the olecranon, just near the first electrode of the Equimetre^TM^ device (Figure 1D), and the right arm electrode (negative) was placed two-thirds of the way down the right jugular groove (Figure 1E). The third electrode was placed cranial and parallel to the second thoracic girth electrode of the Equimetre TM on the left chest. The electrodes were moistened with tap water to maintain electrical conduction and obtain a high-quality ECG signal from both devices. Gel was used for the standard base-apex system.

### 2.3. Data collection

The measurements were recorded by the same operator. The Equimetre^TM^ device was linked through Bluetooth to the Equimetre^TM^ ECG application downloaded from the App store on an iPhone 7 (Apple, USA). The ECG was digitally displayed at a paper speed of 25 mm/s on a smartphone (Figure 1F), and ECG tracing was automatically digitalized by the device. For the standard base-apex method, the ECG tracing was viewed and printed at a paper speed of 25 mm/s with a gain of 40 mm/mV using lead III. Once a satisfactory ECG from each device was determined, a 30 s reading was obtained. One to three readings from each camel were obtained depending on the stress level of the camel. The tracing time setup was simultaneously recorded for both devices at the same time. Moreover, both methods were assessed in terms of time to fix and ECG quality.

### 2.4. Data acquisition

Raw data of ECG were downloaded from Equimetre^TM^ unit in laptop through a specific program supported by the manufacturer (EquimetreUSB_2.0.1 for windows: Arioneo, France) and the data were exported in excel format. Raw ECG data was imported by Kubios software (Kubios Version3.2 for windows: Kubios Oy, Finland) for ECG complexes view and subtraction the none records ECG time before and after ECG reading. All tracings were reviewed for baseline artifacts of P wave, QRS complex and T wave segments were could not be identified. Tracings ECGs were considered acceptable for interpretation if the baseline artifacts were absent for at least 80% (26).

### 2.5. Data analysis

**Figure 2:**
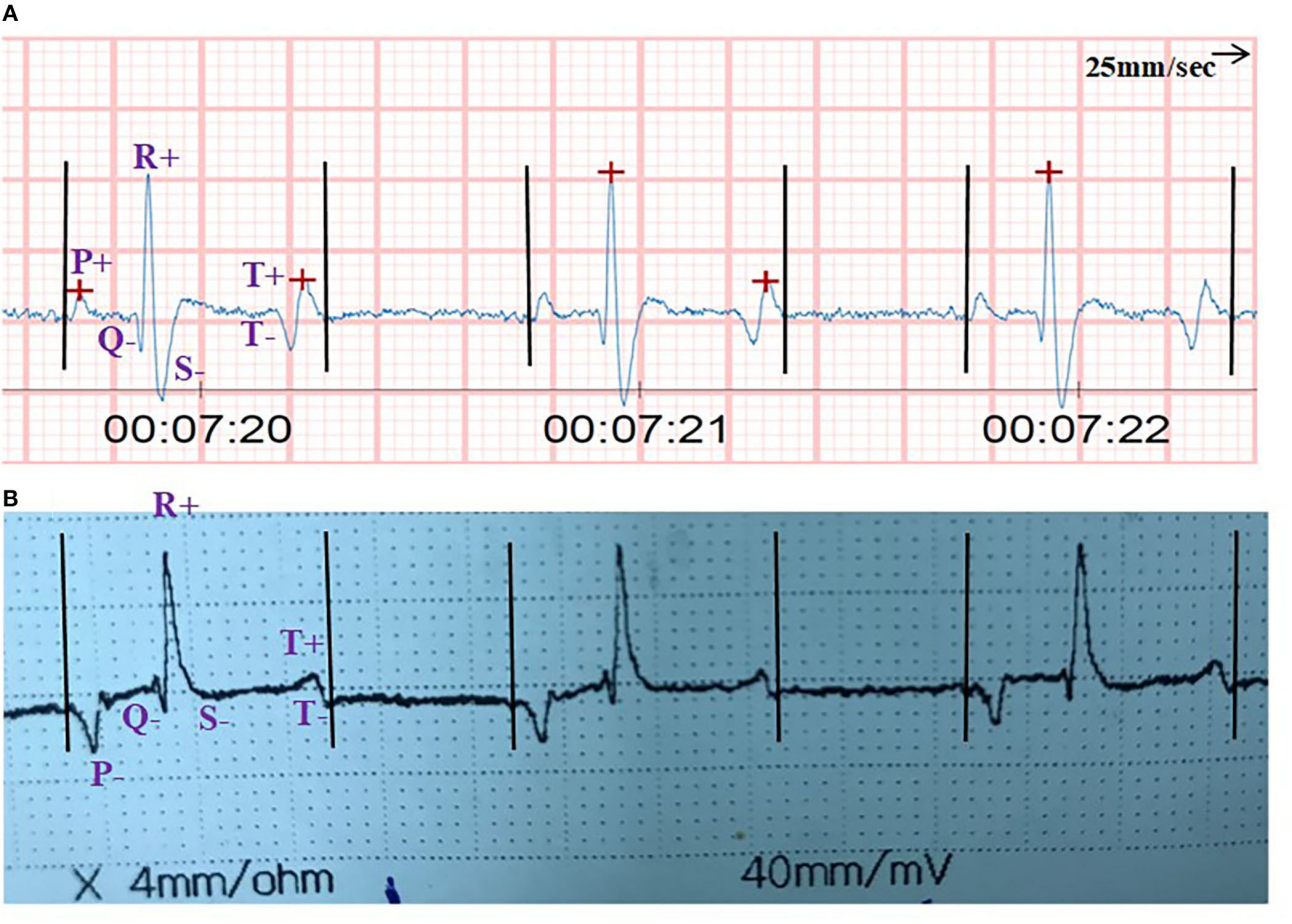
Show ECG tracing from **(A)** Equimetre^TM^ **(B)** Gold standard base-apex for clinical female calf. Camel suffering cloudy eyes referred to King Faisal veterinary teaching hospital. Variation between Equimetre^TM^ and Base-apex system in the complex morphology was noticed, P-Wave showed different polarity (+) in Equimetre^TM^ and (-) in base-apex. Moreover, absence of S-Peak in base-apex system compared to Equimetre^TM^ where S-Peak is present and clear. Additionally, Q-Wave, R-Wave, and T-Wave determined in both devices with respect to the difference between the device in terms of duration and amplitude.

A 15 s reading from the reviewed ECG tracing was selected for the analysis. Each tracing was anonymized and randomized to evaluate the normal ECG variables, including heart rate (HR) in beats per min, by multiplying the number of PQRST complexes from the 15 s trace by 4; P–R interval (s); QRS duration (s); R-R interval (s); Q–T interval (s); S–T interval; P–R segment, and S–T segment (s). Additionally, the amplitudes for the P-peak, Q-peak, R-peak, S-peak, and T-peak (millivolts) and presence of an abnormal complex were recorded. The first and last three complexes from the 15-s ECG tracing were measured for each single measurement, and the average of the measurements over the three complexes was used for analysis. Three trained examiners blinded to the ECG trace identity performed the reading of the trace. The source of the trace was not blinded because of the difference in the trace displayed by each piece of the equipment (Figure 2). All measurements were performed manually (by hand) for both the Equimetre^TM^ and standard ECG without the assistance of ECG interpretation software (19, 24, 26, 27). The data were categorized into three groups: apparently healthy adults, clinical adult cases, and clinical calves (Table 1).

**Table 1:** Details of participated camels.

**Category**	**Sex**	**Age**	**BSC**	**Status**
Apparently healthy adult	4 females and 1 male	8–10 years	3 to 4	Apparently healthy
Clinical adult	1 female and 4 males	7–10 years	2 to 4	2 males have paraphimosis 1 male have inflamed dulla 1 female have ear infection 1 male have open wound on mandible
Clinical calves	2 females	5–6 months	3 to 4	Both have Cloudy eyes

### 2.6. Statistics analysis

All camel categories were described according to age, sex, BSC, and health status. Each measurement using either device (Equimetre^TM^ vs. standard ECG) was first averaged over the three examiners and summarized for each category of camels using the median and range. The correlation between devices was determined using intracluster correlation (ICC) statistics. The agreement between examiners for either device was also assessed using ICC statistics. ICC values were interpreted as having a weak correlation, fair, moderate, good, very good, and perfect agreement for values ≤0.20, 0.21–0.40, 0.41–0.60, 0.61–0.80, 0.81–0.99, and 1, respectively, as described previously (18). All analyses were performed using R software version 3.6.1. Statistical significance was set at a two-sided *p*-value < 0.05.

## 3. Results

ECG traces were obtained from all 12 camels using an Equimetre^TM^ and the standard base-apex system.

### 3.1. Feasibility of ECGs devices

The fixing time of electrodes for the Equimetre^TM^ on camels was estimated to be between 2–3 min, whereas that of the standard base-apex system was 5–6 mindue to the thickness of the camel skin and the grip of the small tip alligator electrodes. Moreover, the standard base-apex system displays the ECG trace immediately after fixing the electrodes, whereas a connection to the mobile app is needed to view the ECG trace of the Equimetre^TM^. Equimetre^TM^ showed stability on ECG tracings with fewer movement artifacts compared with the standard base-apex system. However, the baseline ECG trace from the Equimetre^TM^ showed irregularity compared with the standard base-apex system. A difference in polarity in the ECG complexes was noticed between the devices in P and T waves. The standard base-apex system showed a negative P-wave, whereas the Equimetre^TM^ P-wave was positive. A biphasic T-wave in Equimetre^TM^ showed a negative wave followed by a positive wave, whereas the standard base-apex system started with a positive T-wave followed by a negative T-wave. Furthermore, S-waves were not present in the standard base-apex system in all ECG traces, whereas in Equimetre^TM^, S-waves were present and clear in all ECG traces from all camels.

### 3.2. Heart rhythm

**Figure 3:**
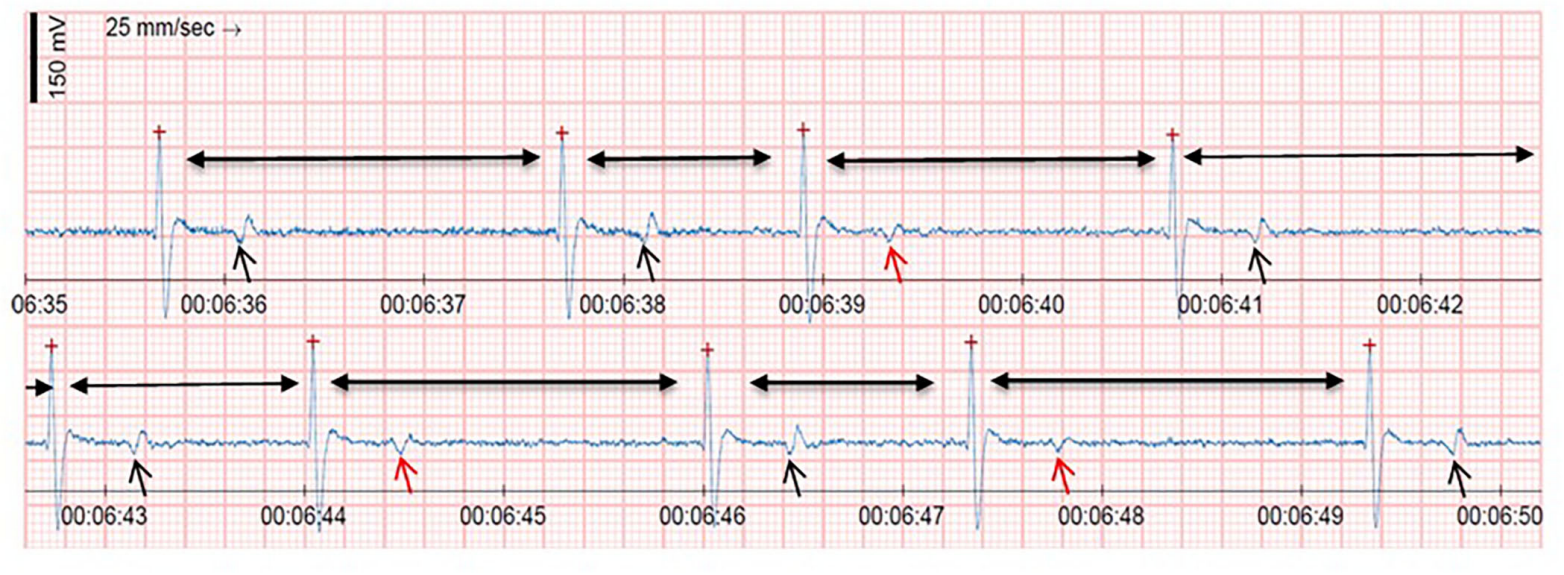
Shows continuous ECG trace from Equimetre^TM^ for clinical male camel suffering paraphimosis referred to King Faisal Veterinary Teaching Hospital. ECG trace revealed heart rate of 36 bpm associated with three junctional premature beats (JPB). Moreover, ECG trace showed a different T-waves polarity in which black arrows showed a normal complex followed by biphasic T-Waves and red arrow showed JPB followed by T^(−)^ waves.

Two camels from the clinical adult category complaining of paraphimosis were detected to have a junctional premature beat (JPB) on continuous ECG tracing using Equimetre TM,; the junctional premature beats seen ranged from 30 to 36 bpm in a total of 15–17 JPB per ECG record. Changes in T-wave polarity were observed in one case of paraphimotic male camel associated with JPB, as shown in Figure 3. The ECG trace showed a normal heartbeat associated with biphasic T-waves, whereas premature junctional beat complexes were associated with negative T-waves. Sinus tachycardia was identified in the normal adult category, with heart rates ranging from 60 to 96 bpm.

### 3.3. Heart rate

The heart rate median and range of each camel category are listed in Table 2 for both devices. The HR agreement over the examiners for the average correlation between standard base-apex and Equimetre^TM^ is shown in Table 3. HR correlation was very good for the apparently healthy camel category, (ICC = 0.90, *P* ≤ 0.0001, 95% = 0.73–0.97), good for clinical adults (ICC = 0.71, *P* = 0.0269, 95% = −0.02 0.95), and moderate for clinical calves (ICC = 0.46, *P* = 0.1265, 95% = −0.37 0.9). The intraclass correlation coefficients between examiners of HR and each device for each category are shown in Table 4. A perfect correlation was observed between examiners of HR in apparently healthy and clinical adult categories for both devices. However, a weak correlation between examiners was observed in the clinical calves category for both devices for HR.

**Table 2:** Summary of ECG measurements for healthy adult, clinical adult, and clinical calves camel.

	**Apparently healthy camel**		**Clinical adult**		**Clinical calves**	
**Measurement**	**Standard base-apex Median (range)**	**Equimetre**^TM^ **Median (range)**	**Standard base-apex Median (range)**	**Equimetre**^TM^ **Median (range)**	**Standard base-apex Median (range)**	**Equimetre**^TM^ **Median (range)**
Heart rate (bpm)	60 (48, 96)	60 (42, 90)	36 (30, 36)	36 (32, 40)	54 (54, 60)	54 (48, 60)
P–R interval (s)	0.20 (0.16, 0.26)	0.13 (0.08, 0.19)	0.20 (0.16, 0.21)	0.18 (0.12, 0.20)	0.19 (0.18, 0.20)	0.14 (0.14, 0.16)
QRS duration (s)	0.11 (0.10, 0.19)	0.12 (0.10, 0.19)	0.12 (0.09, 0.12)	0.13 (0.11, 0.14)	0.10 (0.08, 0.11)	0.10 (0.10, 0.12)
S–T interval (s)	0.28 (0.23, 0.31)	0.30 (0.23, 0.36)	0.31 (0.29, 0.39)	0.35 (0.33, 0.38)	0.29 (0.27, 0.30)	0.32 (0.31, 0.33)
Q–T interval (s)	0.38 (0.34, 0.44)	0.47 (0.38, 0.53)	0.44 (0.44, 0.50)	0.53 (0.51, 0.54)	0.38 (0.38, 0.40)	0.48 (0.47, 0.49)
P–R segment (s)	0.10 (0.08, 0.15)	0.08 (0.05, 0.13)	0.18 (0.10, 0.19)	0.09 (0.07, 0.14)	0.12 (0.11, 0.12)	0.09 (0.08, 0.09)
S–T segment (s)	0.16 (0.06, 0.20)	0.18 (0.11, 0.26)	0.21 (0.21, 0.25)	0.26 (0.24, 0.28)	0.20 (0.19, 0.21)	0.21 (0.20, 0.22)
R–R interval (s)	1.10 (0.68, 1.31)	1.08 (0.70, 1.37)	1.67 (1.32, 1.96)	1.65 (1.46, 1.79)	1.09 (1.01, 1.14)	0.91 (0.63, 1.19)
P-Peak (mv)	−0.27 (−0.30, −0.14)	0.10 (0.03, 0.17)	−0.05 (−0.09, 0.00)	0.13 (0.04, 0.13)	0.07 (0.03, 0.08)	0.09 (0.09, 0.11)
Q-Peak (mv)	−0.40 (−0.87, −0.27)	−0.30 (−0.57, −0.20)	−0.57 (−0.60, −0.27)	−0.36 (−0.51, −0.17)	−0.20 (−0.20, −0.17)	−0.30 (−0.37, −0.30)
R-Peak (mv)	1.34 (0.98, 1.90)	0.84 (0.63, 1.08)	1.41 (1.04, 1.47)	1.50 (1.37, 1.61)	0.75 (0.58, 0.78)	1.06 (1.00, 1.14)
S-Peak (mv)	0.00 (0.00, 0.00)	−0.63 (−0.80, −0.54)	0.00 (0.00, 0.00)	−0.87 (−1.03, −0.81)	0.00 (0.00, 0.00)	−0.72 (−0.80, −0.70)
T-Peak+ (mv)	0.17 (0.00, 0.68)	0.07 (0.00, 0.47)	0.24 (0.21, 0.27)	0.00 (0.00, 0.20)	0.11 (0.07, 0.22)	0.28 (0.20, 0.37)
T-Peak- (mv)	−0.18 (−0.74, 0.13)	−0.31 (−0.64, −0.16)	0.00 (−0.07, 0.00)	−0.23 (−0.29, −0.17)	−0.08 (−0.13, 0.02)	−0.20 (−0.26, −0.20)

Beat per min (bpm); Second (s); Millivolt (mv).

**Table 3:** Agreement between two methods of ECG trace for intraclass correlation coefficient (ICC) and average (AVE) over three examiners for each category.

	**Apparently healthy camel**			**Clinical adult**			**Clinical calves**		

**Variable (AVE)**	**ICC**	***P-*** **value**	**95% CI**	**ICC**	***P*** **-value**	**95% CI**	**ICC**	***P*** **-value**	**95% CI**
Heart rate (bpm)	0.90	< 0.0001	0.73 to 0.97	0.71	0.0269	−0.02 to 0.95	0.46	0.1265	−0.37 to 0.9
P–R interval (s)	−0.54	0.9769	−0.83 to −0.01	0.12	0.3834	−0.65 to 0.80	−0.86	0.9941	−0.98 to −0.32
QRS duration (s)	0.01	0.4874	−0.51 to 0.53	0.45	0.1365	−0.39 to 0.90	0.24	0.2836	−0.57 to 0.84
S–T interval (s)	0.75	0.0007	0.38 to 0.92	0.22	0.2965	−0.58 to 0.83	−0.77	0.9792	−0.96 to −0.04
Q–T interval (s)	0.09	0.3765	−0.45 to 0.59	−0.71	0.9643	−0.94 to 0.09	−0.97	0.9998	−0.99 to −0.79
P–R segment (s)	−0.18	0.7345	−0.64 to 0.38	−0.56	0.9070	−0.91 to 0.33	−0.87	0.9953	−0.98 to −0.36
S–T segment (s)	−0.02	0.5291	−0.54 to 0.51	−0.59	0.9201	−0.92 to 0.29	−0.24	0.6931	−0.81 to 0.62
R–R interval (s)	0.69	0.0023	0.27 to 0.89	0.64	0.0471	−0.14 to 0.94	0.12	0.3794	−0.65 to 0.80
P-Peak (mv)	−0.94	0.9877	−0.98 to −0.83	−0.85	0.9927	−0.97 to −0.28	−0.64	0.9429	−0.93 to 0.20
Q-Peak (mv)	0.42	0.0628	−0.13 to 0.77	0.53	0.0924	−0.30 to 0.92	−0.86	0.9937	−0.97 to −0.31
R-Peak (mv)	−0.51	0.9688	−0.81 to 0.03	−0.15	0.6176	−0.78 to 0.68	−0.82	0.9895	−0.97 to −0.21
S-Peak (mv)	−0.98	0.9886	−0.99 to −0.93	−0.98	0.9769	−1.00 to −0.89	−0.99	0.9958	−1.00 to −0.94
T-Peak+ (mv)	0.00	0.4970	−0.52 to 0.53	−0.75	0.9755	−0.95 to 0.00	−0.66	0.9503	−0.93 to 0.17
T-Peak- (mv)	−0.01	0.5050	−2.19 to 0.69	−0.86	0.9875	−0.56 to 0.38	−0.53	0.9688	−0.36 to 0.11

Second (s); Millivolt (mv): P- value was set for (p < 0.05) of significance^*^.

**Table 4:** Intraclass correlation coefficient (ICC) between examiners and each device for each category.

**Variables (AVE)**	**Devices**	**Apparently healthy camel ECG**			**Clinical adult**			**Clinical calves**		
		**ICC**	***P*** **-value**	**95% CI**	**ICC**	***P*** **-value**	**95% CI**	**ICC**	***P*** **-value**	**95% CI**
HR	Standard	1.00	< 0.0001	1.00 to 1.00	1.00	< 0.0001	1.00 to 1.00	0.07	0.4633	−0.33 to 0.65
	Equimeter^TM^	1.00	< 0.0001	1.00 to 1.00	1.00	< 0.0001	1.00 to 1.00	−0.14	0.6807	−0.39 to 0.51
^*^P–R (s)	Standard	0.53	0.0008	0.20 to 0.84	0.67	0.0025	0.22 to 0.94	−0.09	0.5996	−0.37 to 0.57
	Equimeter^TM^	−0.15	0.8218	−0.34 to 0.21	−0.18	0.8022	−0.37 to 0.29	−0.05	0.5303	−0.35 to 0.61
^*^QRS (s)	Standard	0.02	0.486	−0.25 to 0.39	−0.37	0.9607	−0.46 to 0.07	−0.21	0.7847	−0.41 to 0.42
	Equimeter^TM^	−0.11	0.724	−0.31 to 0.28	−0.47	0.9787	−0.49 to −0.4	−0.32	0.9188	−0.45 to 0.21
^*^S–T (s)	Standard	0.38	0.0134	0.04 to 0.71	0.84	< 0.0001	0.53 to 0.97	−0.01	0.4681	−0.33 to 0.64
	Equimeter^TM^	0.41	0.0090	0.07 to 0.73	0.19	0.1619	−0.17 to 0.66	0.04	0.3955	−0.31 to 0.68
^*^Q–T (s)	Standard	0.28	0.0471	−0.04 to 0.65	0.61	0.0065	0.13 to 0.92	−0.09	0.6049	−0.37 to 0.56
	Equimeter^TM^	0.17	0.1443	−0.13 to 0.56	−0.40	0.6957	−0.47 to 0.16	0.06	0.3634	−0.30 to 0.72
^∧^P–R (s)	Standard	0.66	< 0.0001	0.37 to 0.87	−0.15	0.6903	−0.39 to 0.50	−0.03	0.5124	−0.34 to 0.62
	Equimeter^TM^	−0.08	0.6767	−0.32 to 0.34	−0.11	0.6792	−0.34 to 0.38	−0.01	0.4797	−0.34 to 0.64
^∧^S–T (s)	Standard	0.41	0.0078	0.07 to 0.73	−0.15	0.688	−0.39 to 0.51	0.06	0.4582	−0.33 to 0.65
	Equimeter^TM^	0.61	0.0001	0.31 to 0.84	0.34	0.0707	−0.09 to 0.73	0.09	0.3217	−0.28 to 0.72
^*^R–R (s)	Standard	0.94	< 0.0001	0.87 to 0.98	0.73	0.0008	0.32 to 0.95	−0.02	0.4816	−0.34 to 0.64
	Equimeter^TM^	0.70	< 0.0001	0.42 to 0.88	0.84	< 0.0001	0.59 to 0.96	−0.06	0.5461	−0.35 to 0.60
P-Peak (mv)	Standard	0.15	0.1781	−0.15 to 0.54	−0.44	0.9943	−0.48 to −0.19	0.03	0.4582	−0.33 to 0.65
	Equimeter^TM^	−0.15	0.8202	−0.34 to 0.21	−0.22	0.8746	−0.39 to 0.21	−0.05	0.5437	−0.35 to 0.61
Q-Peak (mv)	Standard	1.00	< 0.0001	1.00 to 1.00	1.00	< 0.0001	1.00 to 1.00	0.02	0.4582	−0.33 to 0.65
	Equimeter^TM^	0.97	< 0.0001	0.94 to 0.99	1.00	< 0.0001	1.00 to 1.00	0.02	0.4582	−0.33 to 0.65
R-Peak (mv)	Standard	0.87	< 0.0001	0.71 to 0.95	0.82	0.0001	0.49 to 0.97	−0.07	0.5663	−0.36 to 0.59
	Equimeter^TM^	0.50	0.0016	0.17 to 0.78	−0.44	0.9995	−0.48 to −0.3	−0.24	0.8211	−0.42 to 0.37
S-Peak (mv)	Standard	1.00	< 0.0001	1.00 to 1.00	1.00	< 0.0001	1.00 to 1.00	1.00	< 0.0001	1.00 to 1.00
	Equimeter^TM^	0.76	< 0.0001	0.52 to 0.91	1.00	< 0.0001	1.00 to 1.00	−0.21	0.7833	−0.41 to 0.42
T-Peak+ (mv)	Standard	0.84	< 0.0001	0.66 to 0.94	−0.33	0.9315	−0.45 to 0.17	−0.03	0.5081	−0.34 to 0.62
	Equimeter^TM^	0.92	< 0.0001	0.81 to 0.97	0.49	0.0078	0.09 to 0.83	−0.03	0.5107	−0.34 to 0.62
T-Peak- (mv)	Standard	0.90	< 0.0001	0.78 to 0.97	0.42	0.0458	−0.06 to 0.87	0.08	0.4582	−0.33 to 0.65
	Equimeter^TM^	0.54	0.0006	0.22 to 0.81	0.17	0.1911	−0.18 to 0.65	−0.02	0.4929	−0.34 to 0.63

^*^Interval in second (s); ^∧^ Segment in second (s); Millivolt (mv): P-value was set for (p < 0.05) of significance^*^.

### 3.4. P–R, QRS, S-T, Q-T, and R-R intervals

The P-R, QRS, S-T, Q-T, and R-R interval medians and ranges for each camel category are shown in Table 2 for both devices. Agreement between examiners for the average correlation between standard base-apex and Equimetre^TM^ for P–R, QRS, S–T, Q–T, and R-R intervals is shown in Table 3. A moderate correlation of the P-R interval average between devices was found for the apparently healthy category (ICC = −0.54, *P* = 0.9769, 95% = −0.83 to −0.01). A weak and very good correlations in P-R interval were observed in the clinical adult (ICC = 0.12, *P* = 0.3834 and 95% = −0.65 to 0.80) and clinical calves (ICC = −0.86, *P* = 0.9941, 95% = −0.98 to −0.32) categories, respectively.

Intraclass correlation for P–R, QRS, S–T, Q–T, and R-R intervals between examiners and each device for each category are shown in Table 4. The standard base-apex showed variation of correlation between examiners for P-R interval measurements were in apparently healthy was good correlation, very good in clinical adult, and week correlation in clinical calves categories. Equimetre^TM^ showed a weak correlation in the P-R interval between examiners in all camel categories.

Correlations of the QRS interval average between devices was noted to be weak in apparently healthy camels (ICC = 0.01, *P* = 0.4874, 95% = −0.51 to 0.53), moderate in clinical adults (ICC= 0.45, *P* = 0.1365, 95% = −0.39 to 0.90), and fair in clinical calves (ICC = 0.24, *P* = 0.2836, 95% = −0.57 to 0.84). The correlation of the QRS interval between examiners was weak for both devices in apparently healthy camels. Moreover, a fair correlation was observed in clinical calf cases for both devices, and for the standard base-apex system in clinical adult cases. However, the Equimetre^TM^ system showed a moderate correlation between examiners for the QRS interval in the clinical adult category.

A good correlation of the S-T interval between devices was observed in apparently healthy camels (ICC = 0.75, *P* = 0.0007, 95% = 0.38 to 0.92) and clinical calves (ICC = −0.77, *P* = 0.9792, 95% = −0.96 to −0.04). However, a fair S–T interval correlation was observed in clinical adults (ICC = 0.22, *P* = 0.2965, 95% = −0.58 to 0.83). A very good correlation between examiners for standard base-apex system in S-T interval measurement was observed in clinical adult cases. However, fair and moderate correlations between examiners were observed in the S-T interval in apparently healthy camels for the standard base apex and Equimetre^TM^, respectively. Moreover, a weak correlation between examiners was observed for the S-T interval in clinical calf cases for both devices and in the Equimetre^TM^ system in clinical adult cases.

A weak Q-T interval average correlation between devices was observed in apparently healthy camels (ICC = 0.09, *P* = 0.3765, 95% = −0.45 to 0.59), good in clinical adults (ICC = −0.71, *P* = 0.9643, 95% = −0.94 to 0.09), and very good in clinical calves (ICC= −0.97, *P* = 0.9998, 95% = −0.99 to −0.79). The intraclass correlation coefficient between examiners for the Q-T interval measurements was fair for both devices in clinical calf cases. Moreover, a fair correlation was noted in Equimetre^TM^ for clinical adults and the standard base-apex in apparently healthy categories. Moreover, a good correlation was observed between the examiners for the Q-T interval from the standard base-apex system in the clinical adult category.

A good correlation was observed for the R-R interval between devices in apparently healthy (ICC = 0.69, *P* = 0.0023 and 95% = 0.27–0.89) and clinical adults (ICC = 0.64, *P* = 0.0471, 95% = −0.14 to 0.94). However, a weak correlation was observed in the clinical calves category (ICC = 0.12, *P* = 0.3794, 95% = −0.65 to 0.80). The intraclass correlation coefficient between examiners for the R-R interval measurement was very good for the standard system in the apparently healthy category and the Equimetre^TM^ system in the clinical adult category. A good R-R interval correlation between examiners was observed in the standard system for clinical adults and Equimetre^TM^ in apparently healthy categories. Moreover, a weak correlation was observed between the examiners for the R-R interval for both devices in the clinical calf category.

### 3.5. P–R and S–T segments

The P-segment median and S-range of each camel category are shown in Table 2 for both devices. Agreement between examiners for the average correlation between the standard base-apex and Equimetre^TM^ for the P–R and S–T segments is shown in Table 3. Week correlation in the average of P–R segment (ICC = −0.18, *P* = 0.7345, 95% = −0.64 to 0.38) and S–T segment (ICC= −0.02, *P* = 0.5291, 95% = −0.54 to 0.51) for both devices in apparently healthy category. Moreover, a moderate average correlation was observed between the devices for the P-R and S-T segments in the clinical adult category (ICC = −0.56, *P* = 0.9070, 95% = −0.91 to 0.33/ICC= −0.59, *P* = 0.9201, 95% = −0.92 to 0.29, respectively). The average correlation of the P–R and S–T segments between devices in clinical calves was very good for the P-R segment (ICC = −0.87, *P* = 0.9953, 95% = −0.98 to −0.36) and fair for the S–T segment (ICC = −0.24, *P* = 0.6931, 95% = −0.81 to 0.62).

The correlations for the P–R and S–T segments between examiners and each device for each category are shown in Table 4. The ICC between examiners for the P–R segment measurement was weak for both devices in the clinical adult and clinical calf categories. Moreover, the correlation between examiners in the apparently healthy category for the P–R segment was strong in the standard system and weak in the Equimetre^TM^ system. A good correlation between the examiners for the S–T segment in Equimetre^TM^ and a moderate correlation in the standard system for the apparently healthy category was noted. However, the correlation for the S–T segment between examiners in the clinical calves category was weak for both devices. The standard base-apex system in the clinical adult category showed a weak correlation for the S–T segment, whereas a fair correlation was observed for Equimetre^TM^.

### 3.6. P, Q, R, S, and T Peaks

Peaks medians and ranges for P, Q, R, S, and T for each camel category are shown in Table 2 for both devices. Agreement between examiners for the average correlation between the standard base-apex and Equimetre^TM^ for P, Q, R, S, and T peaks is shown in Table 3. The average correlation between devices was very good for the P-peak in the apparently healthy category (ICC = −0.94, *P* = 0.9877, 95% = −0.98 to −0.83) and the clinical adult category (ICC = −0.85, *P* = 0.9927, 95% = −0.97 to 0.28). However, the clinical calf category showed a good correlation between devices for the P-peak (ICC = −0.64, *P* = 0.9429, 95% = −0.93 to 0.20). The ICC between examiners for the P-peak measurement was weak for both devices in the apparently healthy and clinical calf categories. Nevertheless, a moderate correlation between examiners in the clinical adult category for the P-peak was observed in the standard system, whereas the Equimetre^TM^ system showed a fair correlation (Table 4).

A moderate correlation was observed between devices for the Q-peak in apparently healthy cases (ICC = 0.42, *P* = 0.0628, 95% = −0.13 to 0.77) and clinical adults (ICC = 0.53, *P* = 0.0924, 95% = −0.30 to 0.92). However, a very good correlation was noted in clinical calves (ICC = −0.86, *P* = 0.9937, 95% = −0.97 to −0.31) in the Q-peak between devices. The ICC between examiners for the Q-peak was perfect for both devices in the clinical adult category and in the apparently healthy category for the standard system. Moreover, Equimetre^TM^ showed a very good correlation between examiners in the apparently healthy category for the Q-peak. Nevertheless, a weak Q-peak correlation between examiners was observed for both devices in the calves (Table 4).

A moderate, weak, and very good correlation of the R-peak average between devices was noted in apparently healthy camels (ICC = −0.51, *P* = 0.9688, 95% = −0.81 to 0.03), clinical adult (ICC = −0.15, *P* = 0.6176, 95% = −0.78 to 0.68), and clinical calves (ICC= −0.82, *P* = 0.9895, 95% = −0.97 to −0.21). The correlation between examiners for the R-peak was very good for the standard system in apparently healthy and clinical adults. However, in Equimetre^TM^ a moderate correlation between examiners in the apparently healthy and clinical adult categories for R-peak was observed. Nevertheless, a weak R-peak correlation between examiners was observed for both devices in calves (Table 4).

A very good correlation was noted between devices for the S-peak in apparently healthy (ICC = −0.98, *P* = 0.988, 95% = −0.99 to −0.93), clinical adult (ICC = −0.98, *P* = 0.9769, 95% = −1.00 to −0.89), and clinical calves (ICC = −0.99, *P* = 0.9958, 95% = −1.00 to −0.49). The correlation between examiners for the S-peak was perfect for the standard system for all camel categories. However, in Equimetre^TM^ a perfect correlation was observed between examiners in the clinical adult category for the S-peak. Nevertheless, a good S-peak correlation between examiners was observed in apparently healthy adults, and a fair correlation was observed in clinical calves (Table 4).

A good correlation was observed between devices for the T^+^-peak in clinical adults (ICC = −0.75, *P* = 0.9755, 95% = −0.95 to 0.00) and clinical calves (ICC = −0.66, *P* = 0.9503, 95% = −0.93 to 0.17). However, there was a weak correlation between devices in the apparently healthy category (ICC = 0.00, *P* = 0.4970, 95% = −0.52 to 0.53). The correlation between examiners for the T^+^-peak was very good for both devices in healthy individuals. However, a weak correlation between examiners in the clinical calves category for the T^+^-peak was observed. Nevertheless, a moderate T^+^-peak correlation between examiners was observed in the Equimetre^TM^ system, whereas the standard system was fair in clinical adult cases (Table 4).

The correlation between devices for T^−^ -peak was weak in apparently healthy adults (ICC = −0.01, *P* = 0.5050, 95% = −2.19 to 0.69), very good in clinical adults (ICC = −0.68, *P* = 0.9875, 95% = −0.56 to 0.38), and moderate in clinical calves (ICC = −0.53, *P* = 0.9688, 95% = −0.36 to 0.11). The correlation between examiners for the T^−^-peak was very good in apparently healthy subjects for the standard system. Moreover, a moderate correlation between examiners was observed for Equimetre^TM^ in the apparently healthy category for T^−^-peak and the standard system in the clinical adult category. Nevertheless, a weak T^−^-peak correlation between examiners was observed in clinical calf cases only for both devices (Table 4).

## 4. Discussion

There is increasing interest in the veterinary field to use mobile health and wearable devices to monitor vital signs (heart rate, heart rate variability, and respiratory rate) in field and farm conditions, with the capability of identifying physiological and pathological correlations with stress, distress, and other emotional states (10, 12). Visualizing the underlying electrical activity of the camel's heart using ECG is critical for determining the type of arrhythmia present (28, 29).

Our study findings proposed that: (1) applying the Equimetre^TM^ fitness health tracker was feasible in camels and provided an acceptable method for ECG recording; (2) adequate ECG tracings for interpretation could be obtained from Equimetre^TM^; (3) there was a moderate to very good correlation between the Equimetre^TM^ and standard system for the HR; and (4) acceptable measurements of waves, intervals, segments, and peaks could be obtained from Equimetre^TM^. This suggests the practical usefulness of Equimetre^TM^ for camels.

The stability and minimum movement artifacts in Equimetre^TM^ compared to the standard base-apex complex in the present study were in agreement with (27), who suggested that wearable devices reduce the equipment needed on and around the horse compared to standard ECG, and is therefore potentially less stressful. However, fixing the electrodes on the camel was difficult using the standard base-apex method, as expected from high skin tension while the camel was in a sitting position vs. the small tips of alligator electrodes, which might cause loss of grip and interrupt the ECG recording. Nevertheless, this problem was not observed in the Equimetre^TM^ as a result of the tight electrode fixed to the animal body by the girth. Interestingly, the detection of junctional premature beats in two clinical adult camels in the present study from continuous Equimetre^TM^ ECG recordings that were not present on standard base-apex ECG traces or could be missed on a monitor was in agreement with a previous study on horses using Equimetre^TM^ for detecting arrhythmia in continuous ECG recordings during exercise (24). To the best of our knowledge, there have been no reports in the literature regarding junctional premature beats in camels.

Considering that the heart rate of the mature camel ranges from 28 to 50 beats per min (29, 30), the correlation between Equimetre^TM^ and the standard base-apex system of heart rate was good to very good in adult camels for both devices, and the agreement was perfect depending on the examiners. This demonstrated the usability of Equimetre^TM^ as a wearable ECG device for measuring HR in adult camels, similar to horses (19). A few adult camels showing an increase in HR were all from apparently healthy categories in the present study, which may be explained by the stress at the time of ECG recording. The incompatibility between the two devices in calves for the measurement of HR may be explained by the difficulty of fixing the electrode and girth around the chest in these animals and, consequently, the difficulty of obtaining compatibility in the work of the heart during recording.

The variability in the ECG measurements between devices and examiners of intervals, segments, and peaks could be due to the differences in electrode number, type, and placement in the animals. The weak to moderate correlation in the P-R interval and P-R segment measurements between the two devices in the adult camels in this study might be due to the difficulty in identifying P waves by examiners for Equimetre^TM^ in most ECG traces, because the agreement between examiners for the P-R interval was weak, unlike the standard base-apex system. Although Equimetre^TM^ had a weak correlation with the P-R interval in adult camels, the agreement between the standard base-apex and Equimetre^TM^ was very good for calves in the presence of clear P waves on ECG traces. Indeed, the small circumference of the chest in calves and the large electrode size of Equimetre^TM^ might enable heart signals to be recorded by the electrodes more efficiently, and the P wave is clearly identified. Conversely, several studies on smartphone ECG devices in horses and cows have found no agreement on P-wave polarity between the standard base-apex and smartphone ECGs, presumably because of the small dipolar electrodes of the smartphone ECG and the placement of the device on the chest (18, 26, 27).

In essence, the fair to weak correlation for QRS, S-T, and Q-T intervals in most camel categories in the present study could be explained by the difference in the determination of QRS, S-T, and Q-T intervals between examiners for both devices. However, moderate to very good correlations of QRS, S-T, and Q-T intervals between examiners suggested that Equimetre^TM^ may still be acceptable for measuring ECG complex variables in camels, which are difficult to compare due to the lack of available data in camel ECG measurements, to the best of our knowledge. In addition, measurements from ECG Equimetre^TM^ tracings should be carefully evaluated, and future studies should be performed to validate the ECG reliability. This is in agreement with a previous report (19). The similar QRS interval readings in the calf camel category for the standard base-apex and Equimetre^TM^ devices in the present study were in agreement with previous reports (31). Ironically, a good correlation in the R-R interval between devices and examiners in adult camels in the present study was found to be reliable, suggesting that Equimetre^TM^ can be useful for the measurement of the R-R interval as the difference in most cases was minimal. Similarly, a previous study reported the utility of a smartphone ECG for R-R interval measurement in healthy horses (19). The differences in the R-R interval in calf camels between the two devices could be explained by the inappropriate girth size in Equimetre^TM^ for these animals.

A weak to moderate correlation between devices for the S-T segment in the current study may be explained by the lack of the S-wave in standard base-apex monitoring compared to Equimetre^TM^. This is in contrast with a previous case report by (31) on camel calves with atrioventricular block where the S-wave was present in the standard base-apex. This suggests that Equimetre^TM^ may be useful for measuring this complex region. The difficulty of determining the P-peak, including abnormal P waves for Equimetre^TM^ in adult camels in the present study and its variation among camel categories, led to a lack of correlation between the examiner and devices; as described above, examiners were unable to distinguish the P-wave. Furthermore, there was a weak correlation of the R-peak in the present study among camel categories and between the two recording methods. This is not unexpected because the difference in fixation of the electrodes in both devices and possibly insufficient wetting of Equimetre^TM^ electrodes (as water acts as a conductor for heart electrical impulse), may have a role in the intensity of the ECG recording.

There was no correlation between T^+^-peak and T^−^-peaks in the present study between the two recording methods in all camel categories. Nevertheless, the correlation between examiners ranged from very good to weak in most camel categories. The compatibility between the examiners in determining the negative and positive T-peaks can be explained by its good clarity in the camel ECG by both devices, while the reason for the lower correlation between the two devices in determining the T-peak may be explained by the difference in polarity displayed by the ECG on these devices. Based on comments from our examiners, the main challenge with the P-wave, T-wave, P-R, Q-T, and S-T interval measurements was identifying where the P-wave and T-wave started and ended due to undulation in the baseline of standard base-apex and Equimetre^TM^ devices in dromedary camels. Moreover, S-T was determined in the standard base-apex owing to the lack of S-waves. These findings are similar to what has been previously described in studies comparing standard base-apex and other wearable devices (27, 32), in which they noticed some degree of variability in the identification of waveform and polarity between observers and devices. Additional studies using larger numbers of healthy and diseased camels should be conducted to confirm and interpret these observations.

One of the main limitations of this study is the small number of camels included and the difference in breeds, age, sex, and body weight of the camels. Additionally, the healthy camel group may have been under the influence of stress during the recording due to the unfamiliarity of procedures and operators. Unlike for clinical cases, these healthy animals not used to be handled by different animal caretakers.

In conclusion, this study demonstrated acceptable ECG measurements between standard base-apex and Equimetre^TM^ devices. This suggests that Equimetre^TM^ could be a useful device in camels for initial electrocardiograph examination because it is portable and requires no training, especially in remote areas such as deserts. However, Equimetre^TM^ is not a substitute for a standard base apex. Further studies are required to verify the ability of the Equimetre^TM^ to detect arrhythmias and heart performance in a large population of camels. Additionally, future studies are needed to determine the capabilities of Equimetre^TM^ to assess camel heart performance in motion and race as well as other parameters.

## Data availability statement

The original contributions presented in the study are included in the article/supplementary material, further inquiries can be directed to the corresponding author.

## Ethics statement

The electrocardiogram was done without giving any treatments or causing harm to the animal, and the camels used in the present study were among the camels that visit the Veterinary Teaching Hospital, where they are routinely examined with the approval of the animal breeder.

## Author contributions

TS and TA: contributed equally to study design, execution, data analysis, and manuscript construction. AA and MA-A: contributed to data analysis and critically revised manuscript. All authors contributed to the article and approved the submitted version.
